# Exploring the potential solutions to the criticisms of positive psychology: But can the bold, idealistic visions of positive psychologists survive real-world scrutiny?

**DOI:** 10.3389/fpsyg.2025.1511128

**Published:** 2025-04-24

**Authors:** Llewellyn Ellardus van Zyl

**Affiliations:** ^1^Psynalytics | AI Driven People Analytics, Eindhoven, Netherlands; ^2^Optentia Research Unit, North-West University, Vanderbijlpark, South Africa; ^3^Department of Human Resource Management, University of Twente, Enschede, Netherlands

**Keywords:** criticisms of positive psychology, critiques of positive psychology, participatory action research, solutions, third wave positive psychology

## Abstract

Positive psychology has faced growing criticism regarding its scientific foundations and applied impact. To encourage constructive dialog, this study employs a Participatory Action Research (PAR) approach to co-create potential ways to address the critiques of positive psychology. By engaging positive psychological practitioners and scholars, we investigate their perspectives on addressing the discipline’s theoretical, methodological, and practical challenges and critically evaluate the viability of these solutions. Purposive sampling gathered data from 213 positive psychology researchers and practitioners. Participants were familiarized with the main criticisms through a participatory online workshop and then engaged in small group discussions to generate potential solutions to such. Content analysis identified 16 themes and 37 categories of proposed solutions. While the proposed solutions showed promise, some appear idealistic given academic realities. This participatory study empowers positive psychologists to actively shape the evolution of their field through ongoing dialog, reflective co-creation and knowledge generation.

## Introduction

Positive psychology has emerged as a rapidly expanding subdiscipline within psychology, focusing on the scientific study of optimal human functioning (Wang et al., 2023). While positive psychology has made significant contributions to our understanding of wellbeing, it has faced growing criticism regarding its credibility as a science and usefulness as an applied discipline (Van Zyl and Rothmann, 2022). Critics have raised concerns about positive psychology’s philosophical assumptions, theoretical coherence, research methods, cultural biases, and potential for harm (Van Zyl and Rothmann, 2022; van Zyl et al., 2023). For instance, critics contend that positive psychology lacks a coherent theoretical foundation with clear definitional boundaries (Joseph, 2015), suffers from confirmation bias and an overreliance on Western cultural assumptions (Christopher and Hickinbottom, 2008), employs poorly developed self-report measures and “quick and dirty” research practices (Coyne and Tennen, 2010), and makes exaggerated and unsupported claims about increasing wellbeing (Fernández-Ríos and Novo, 2012). These ongoing criticisms are exasperated by broader credibility issues facing general psychology stemming from failures to replicate seminal studies, incidents of academic fraud, and questionable research practices which signaled a potential “crisis of confidence” in the discipline’s scientific integrity (Efendic and Van Zyl, 2019; Nelson and Slife, 2017; Nosek et al., 2015). These limitations arguably constrain positive psychology from fully delivering on its aims of facilitating flourishing (Lomas et al., 2021; Van Zyl et al., 2024).

To consolidate the contemporary critiques and criticisms of positive psychology, van Zyl et al. (2023) conducted a systematic review identifying 117 distinct criticisms that clustered into six broad themes. They indicated that critics argued that:

(1) Positive psychology *lacked proper theorizing and conceptual thinking*. Critics argued that positive psychology lacked a unified and cohesive metatheory that grounds the philosophy foundations of the science, and failed to provide clear criteria for conceptualizing and approaching positive psychological phenomena.
(2) Positive psychology was *problematic regarding the measurement of positive psychological constructs and employed poor methodologies* to investigate its claims. Detractors argue that the field relies heavily on poor measurement tools, cross-sectional designs, and neglects robust methods such as experimental designs and qualitative approaches. This overreliance on specific methodologies has been viewed as a limitation that potentially compromises the generalizability and validity of the findings.
(3) Positive psychology was seen as *a pseudoscience that lacks evidence for its claims and showing poor replicability*. Critics contended that positive psychology presented claims unsupported by empirical evidence, that it exaggerated the benefits of its interventions, that it was rife with confirmation bias, and that its findings could not be replicated. The inability to consistently replicate results has raised questions about the scientific credibility of the field, casting doubt on the reliability and validity of its claims.
(4) Positive psychology *lacked novelty and self-isolated itself from mainstream psychology*. Critics argued that positive psychology brought nothing new to the proverbial table and that it wilfully created a fictitious divide between “negative” psychology and the study of “optimal human functioning” as a means to justify its existence. Critics argue that the field merely repackages existing concepts and theories from other domains of psychology into new jackets and call it a unique contribution. This perceived lack of originality has led to concerns about the field’s relevance and contribution to the broader discipline.
(5) Positive psychology was *a decontextualized neo-liberal ideology that causes harm*. Critics classified positive psychology as a neo-liberal ideology that positioned Western values as “universal,” neglected the role of culture and context in the creation of its theories, pathologized “normal human behavior,” and also marginalizing vulnerable and underrepresented groups, by reinforcing stereotypes. This creates stigma and causes harm. This implies that the field overlooks sociocultural factors and places excessive responsibility of personal happiness on individuals themselves which potentially leads to victim-blaming and thus neglecting systemic issues. This ideological underpinning has raised concerns about the potential harmful implications of positive psychology’s approach.
(6) Positive psychology was a *capitalistic venture*. Critics perceived the field as being a capitalist venture that is driven by commercial interests rather than scientific rigor. This critique suggests that the field may be influenced by profit-driven motives that potentially compromises its objectivity and scientific integrity.

While not all criticisms are equally compelling, this consolidation of critiques highlights the need for ongoing reflection and discussion to strengthen the field. However, thoughtful engagement with these criticisms alone won’t stop the field from fading into another psychological fad and simply acknowledging its limitations will not automatically strengthen the discipline (Fernández-Ríos and Novo, 2012). Active and collaborative discourse is needed to translate these critiques, criticisms and limitations into constructive reforms that can facilitate the development of a more rigorous, socially responsible, inclusive, and practically relevant science that can enrich our understanding of wellbeing and optimal human functioning (Lomas et al., 2021; Ivtzan and Lomas, 2016).

The present study aimed to contribute to this discourse by adopting a participatory action research approach (PAR) to collaboratively engage positive psychology practitioners, and scholars in reflecting on the discipline’s criticisms and co-creating solutions to address its theoretical, methodological, and practical limitations in an applied manner. PAR is an applied research methodology that focuses on collaboration and active involvement between researchers and participants as a process of knowledge creation through self-inquiry, meaning-making and social change, rather than an extractive data collection process from passive subjects (Kindon et al., 2007). It aims to address real-world problems, empower participants, and promote social change through a participatory and democratic process of co-creation (Kindon et al., 2007).

The PAR methodology does not intend to address scientific criticisms directly (which typically requires empirical evidence and experimentation), but rather it creates a platform for a wide array of participants to share their views on possible solutions to these problems. In essence, PAR does not provide definitive answers to complex scientific problems, but rather creates a space where positive psychological researchers and practitioners could deliberate on potential strategies which could be employed to grow the field. This approach thus allows one to capture the perspectives, thoughts and proposed responses of positive psychology insiders regarding how to constructively respond to the criticisms facing the field. Further, this approach mirrors positive psychology’s aims of fostering self-reflective growth through collective engagement with different perspectives (Velasquez-Mantilla, 2022). By actively involving positive psychology practitioners and scholars in reflecting on the critiques, criticisms, and limitations of the field, we create an inclusive, pragmatic, and empowering platform for inquiry, where proposals to strengthen the discipline can be co-created and readily implemented (Velasquez-Mantilla, 2022).

As such, the purpose of this study was to co-create potential ways to address the criticisms and critiques of positive psychology by eliciting perspectives from positive psychological practitioners and scholars. Specifically, it aimed to reflect positive psychological scientists and -practitioners’ responses/thoughts about the criticisms and their ideas as to how to address them. Through an active, participatory online workshop hosted by the European Network of Positive Psychology (ENPP), small groups reflected on the critiques and generated potential ways to approach solutions, which were analyzed using conventional content analysis to derive actionable recommendations. This paper presents proposed solutions and discusses their potential and challenges for strengthening positive psychology as a science and a practice domain.

## Research methods

### Research approach

This study employed a qualitative, descriptive design to gather perspectives of positive psychological practitioners and scholars on addressing the criticisms and critiques of positive psychology. A PAR approach was employed, emphasizing collaboration between the researchers and participant stakeholders, to reflect upon the discipline’s limitations and to co-creating solutions for strengthening positive psychology as a science and practice domain. PAR was deemed appropriate as it focused on “identifying practical solutions to issues of pressing concern” (Bradbury, 2015, p.1). In essence, PAR aligns well with positive psychology’s humanistic values and orientation toward growth, empowerment and leveraging human strengths in a collaborative manner. It involves a process of active orientation, planning, acting, observing and self-reflection to generate practice solutions to complex problems (Bradbury, 2015). According to Chevalier and Buckles (2019) this approach could afforded several potential benefits for addressing the main research question:

1) *Empowering Stakeholders and Practitioners*. PAR empowers positive psychology practitioners and scholars as key stakeholders by giving them an active voice and involving them directly in the knowledge generation process (Chevalier and Buckles, 2019). Rather than having changes imposed top-down, PAR allows for grassroots participation in critically reflecting on limitations and co-creating potential routes toward solutions that are grounded in their real-world experiences.
2) *Fosters Inclusive Dialog and Involves Diverse Perspectives.* The participatory nature of PAR facilitates an inclusive dialog by bringing together diverse perspectives from various positive psychology scholars and practitioners. This mitigates insular groupthink by opening up the discourse to different viewpoints, expertise, and voices that may be marginalized in traditional research approaches (Chevalier and Buckles, 2019).
3) *Enhances Transparency and Facilitates (Critical) Self-Reflection*. PAR’s cyclical process of collaborative inquiry, action, and reflection cultivates an ethos of transparency within the sample group of positive psychologists. Openly acknowledging criticisms and actively engaging with them through PAR demonstrates a commendable commitment to critical self-reflection and a willingness to evolve as a field.
4) *Grounds Proposed Ways to Solutions in Practical Realities*. By involving seasoned practitioners and scholars, the routes to these solutions proposed through PAR are more likely to be grounded in practical realities which incorporates the contextual challenges faced in researching and applying positive psychology in real-world contexts (Chevalier and Buckles, 2019). This enhances the ecological validity and feasibility of the recommendations compared to the prescriptive instructions / suggestions from theorists and critiques who may be unfamiliar with the realities of implementation (Chevalier and Buckles, 2019).
5) *Builds Commitment to and Ownership of the Routes toward the Solutions.* The democratic, bottom-up process employed via the PAR approach, inherently builds commitment to and ownership between positive psychology stakeholders in the eventual implementation of these solutions. Having participated in co-creating recommendations, it increases their buy-in and personal investment in their implementation.
6) *Facilitates Transformative Change.* By democratizing the generation of knowledge production and fostering a sense of collective empowerment PAR helps to facilitate transformative change. The community’s engagement in this process can catalyze momentum for positive psychology to evolve in a rigorous and socially responsive manner.

These generated solutions to the field’s critiques were followed by a collaborative qualitative, content analysis to derive meaning from the discussions.

### Participants and procedure

A purposive sampling strategy was used to recruit participants (*N* = 213) via the ENPP’s professional network for the study. Participants were self-identified positive psychological practitioners, scholars and students as well as individuals broadly interested in positive psychology as a practice/research domain. As summarized in Table 1, the majority of the participants identified as Female (57.75%), and most participants were from the United Kingdom (26.29%), Türkiye (10.80%), South Africa (7.98%), Netherlands (6.10%) and Germany (4.69%). Participants were invited via the ENPP’s mailing list to register for an online workshop related to the critiques and criticisms of positive psychology. Invitations were sent twice over a period of 6 weeks. Upon clicking the registration link, participants were redirected to a landing page which informed them of the nature and purpose of the workshop, the PAR approach which would be employed, and how data was to be reported/used. Potential participants were also requested to read the van Zyl et al. (2023) paper before the day of the workshop. For privacy, no identifiable demographic- or personal information of participants was captured beyond their gender and current country of residence.

**TABLE 1:** Demographic details of participants (*N* = 213).

Factor	Frequency	Percentage (%)
**Gender**		
Female	123	57.75
Male	53	24.88
Other	37	17.37
**Country of residence**		
Australia	2	0.94
Austria	3	1.41
Belgium	1	0.47
Brazil	3	1.41
Bulgaria	1	0.47
Canada	3	1.41
Côte d’Ivoire	1	0.47
Croatia	2	0.94
Czechia	1	0.47
Denmark	3	1.41
Ecuador	2	0.94
Finland	3	1.41
France	4	1.88
Georgia	2	0.94
Germany	10	4.69
Greece	3	1.41
Hong Kong	1	0.47
Hungary	1	0.47
India	4	1.88
Indonesia	1	0.47
Ireland	3	1.41
Italy	5	2.35
Jamaica	1	0.47
Korea, Republic of	1	0.47
Lithuania	2	0.94
Lithuania	1	0.47
Malta	1	0.47
Mexico	2	0.94
Netherlands	13	6.10
New Zealand	1	0.47
Norway	4	1.88
Pakistan	1	0.47
Poland	1	0.47
Portugal	4	1.88
Quatar	2	0.94
Romania	1	0.47
Singapore	1	0.47
Slovenia	1	0.47
South Africa	17	7.98
Spain	9	4.23
Sweden	1	0.47
Switzerland	2	0.94
Thailand	1	0.47
Turkey	23	10.80
United Kingdon	56	26.29
United States	7	3.29
Viet Nam	1	0.47

The online PAR workshop was facilitated by three individuals and lasted approximately 3 h. The PAR workshop was comprized of three parts. First, participants were oriented to an online lecture of approximately 45 min centered around the 117 criticisms and critiques of positive psychology as summarized in van Zyl et al.’s (2023) review. Second, participants were randomly classified into six small breakout rooms and tasked with reflecting on one of the six major themes of criticisms and generating constructive solutions to address such. Groups documented their discussions and presented key problems, reflections, and proposed solutions in PowerPoint format. This process lasted approximately an hour. Finally, the six groups presented a summary of their solutions to the larger forum, which was then open for debate and discussion to further expand upon each point. The workshop concluded with a reflective discussion on the collaborative process. The 3 h workshop produced conversations centered around proposed solutions to criticism themes which were analyzed and classified.

### Data analysis

The transcribed discussion data were analyzed using an inductive approach through conventional content analysis (Hsieh and Shannon, 2005). Given the participatory nature of the workshop, the discussion content had already undergone an iterative process of refinement and feedback, where participants refined their viewpoints and collaboratively established strategies during the session. We collated these matured, consensus-driven outputs from the discussion by summarizing key thoughts, reflections, and potential solutions articulated across the six groups. This process not only captured the final version of their ideas but also preserved the depth and richness of the collective dialog (Fine and Torre, 2021).

In alignment with core PAR principles, this approach to the analysis honored the co-created nature of the data and emphasized the importance of iterative knowledge building (Fine and Torre, 2021). Initial codes were generated directly from the recorded discussions, ensuring that the emerging themes exactly reflected the participants’ authentic perspectives (Fine and Torre, 2021). These codes were subsequently consolidated into broader categories and themes through a systematic process of integration, whereby overlapping ideas were merged and discrepancies resolved to form a coherent thematic framework (Fine and Torre, 2021; Hsieh and Shannon, 2005). This method not only enhanced the rigor of the analysis but also reinforced the value of participatory dialog as the foundation for actionable insights in positive psychology.

## Results

The content analysis yielded 37 categories of proposed solutions, classified into 16 broad themes across the six themes of criticism in positive psychology. (c.f. Table 2 for a summary of the proposed solutions and their respective categories).

**TABLE 2:** Summary of the proposed solutions to the criticisms and critiques of positive psychology.

No	Criticism	Proposed solutions	Categories of proposed solutions	Example quote
1	Lack of proper theorizing and conceptual thinking	Incorporate diverse perspectives	• Diverse philosophical approaches • Insights from other disciplines • Multidisciplinary collaboration	“We can learn a lot from other philosophical approaches like Stoicism and Buddhism…(sic) disciplines like political sciences and evolutionary biology to help us understand people better”
		Develop holistic meta-theories	• Synthesize holistic theories • Identify linkages	“I think we need something like a meta-theory for wellbeing that pulls everything we’ve done together into one coherent framework. Kinda (sic) like Eriksons theory on human development and how it pulls in psycho-social development stuff with like personally development etc.,”
		Bottom-up approaches and consensus building	• Bottom-up approaches • Consensus on terminology	“…it (definitions of wellbeing) is different from person to person not just country to country, so we need to build models that define wellbeing from the person’s own perspective…” “Academics need to be consistent with the words they use to describe things. One person calls it grit, the other calls it tenacity, the other calls it hardiness… this is confusing”
		Collaborative theory building	• Distinguish academic and practical focuses	“..there needs to be some collaboration between theory and practice because they (academics) are too concerned with things that don’t matter to us (practitioners) and vice versa. They want to explain stuff but we need to do things… we need tools and they need our insights…”
2	Problems with measurement and methodology	Expand methodological approaches	• Alternative assessment tools • Balance objective and qualitative data • Assess collective experiences • Narrative approaches	“Surveys and interviews by themselves cant capture nuances of peoples understanding of wellbeing. Like, we need new types of tools and things like story telling approaches and more mixed method research…. We also need more collective assessments and perspectives on things like wellbeing, not just based on the person…”
		Promote open science practices and data sharing	• Publish non-significant results • Embrace open science practices	“Publish results that aren’t significant and make it open so people can see for themselves”
		Control for social biases and respondent reactivity	• Minimize social desirability • Ensure respondent accuracy	“We should do what the personologists do and start to use more subtle indirect measures to control for social desirability when they complete assessments”
3	Issues with replication and evidence	Improve public science communication	• Educate public on science • Promote responsible media reporting • Establish ethical guidelines	“Academics have a duty to communicate their findings to the public in a responsible manner and the public must be trained to understand what the science actually means so they don’t buy snake oil”
		Centralize communication	• Professional societies publish comprehensive evidence	“There should be a unified platform through which we can communicate things. Like the ENPP can use their website as the basis to communicate research to others. Other professional societies like EAWOP could also help.”
		Actively engage with critics	• Test and communicate on pseudoscience claims • Develop solutions collaboratively	“Look, we do need the critics and we have to engage with them otherwise we stay in a closed loop, and we have to provide evidence that we aren’t a fly by night science”
4	Lack of novelty and self-isolation	Bridge divides through collaboration	• Clarify differences from mainstream psychology • Offer alternative explanations • Explore interplay between perspectives	“.. And a systemic effort is needed to clarify our brand to the outside world, so that we can drop the title of “not focusing on the negative “… we just provide a different lens through which to understand things but its also our responsibility to engage with different domains so we can see where and how we complement each other…”
5	Positioning as decontextualized ideology	Enhance cultural responsiveness	• Test cross-cultural applicability • Build bottom-up theories • Prioritize contexts	“Our theories need to be flexible to accommodate different perspectives from different cultures… we need something like a cross-cultural positive psychology”
		Adopt social responsibility ethos	• Acknowledge societal implications • Focus on diverse populations • “Do no harm” principle	“We can’t just focus on the WEIRD contexts, we have to start focusing on other contexts and stop ignoring broader societal issues as what we are currently doing can cause harm”
		Balance positive and negative	• Explore negative aspects • Avoid one-sided perspectives	“…like the paper from Gerben and Ernst that presented a more unified view of how mental illness and mental health work together. That’s a major step in the right direction…”
		Ensure intervention quality	• Tailor to individual • Formalize training and standards	“With AI we can now do things like present hyper-personalized results to people and I think we are not far off from creating hyper personalized interventions that are completely tailored to each individual”
6	Critiquing as capitalistic venture	Balance access and expenses	• Offer free basic services • Open access initiatives	“It’s easy… we have like a freemium type model, where we can provide like general interventions for free, but the more personalized things need to be paid… we can also make sure that any public funded intervention’s content is made public so we can all learn and grow together”

### Criticism 1: lack of proper theorizing and conceptual thinking

#### Incorporate diverse philosophies and insights from other disciplines

Participants proposed that positive psychology should expand its philosophical foundations and theoretical perspectives by adopting different philosophical perspectives and incorporating insights from other fields. For instance, exploring the relevance of Stoicism and Buddhism, to positive psychology can provide valuable insights into understanding how meaningful life experiences are created. By incorporating diverse philosophical approaches, positive psychology can gain a more comprehensive view of human wellbeing. Further, participants state that positive psychology can enhance its theoretical foundations by incorporating insights from other approaches or fields, such as quantum physics, complexity theory, political science, personology, and evolutionary biology, to enrich its understanding of the human condition. Multidisciplinary collaboration was also viewed as an opportunity to develop more holistic, integrated theories encompassing different aspects of wellbeing. By drawing from these fields and philosophies positive psychology can enrich its understanding of the human condition and broaden its perspectives on the nature, development and importance of positive phenomena.

#### Develop holistic meta-theories integrating different approaches

Relatedly, participants highlighted the need for developing overarching meta-theories in positive psychology that provides a holistic framework that ties its different theories, frameworks and models together. Suggestions involved identifying points of overlap and linkage between theories to work toward an integrated understanding of wellbeing and optimal functioning. For instance, connecting evidence-based insights from positive psychology interventions to broader theories of human needs and development. Participants noted that a single unifying theory may not be feasible or helpful, but continually developing and refining meta-theories can strengthen coherence in the field.

#### Employ inclusive bottom-up approaches to theory building and creating consensus

Bottom-up, inductive approaches to theory development were proposed by participants as a means to make positive psychology more inclusive and grounded in real-world lived experiences. This involves collaborating with community members, practitioners and participants to co-creating conceptual frameworks using qualitative methods versus imposing pre-determined models. As one participant explained, “People should have a voice in defining what wellbeing and thriving means in their context rather than having an expert viewpoint imposed.” Further, participants indicated that consensus in the language used to describe experiences is essential for effective communication and collaboration within positive psychology. Establishing common terminology and definitions can enhance clarity and facilitate a more coherent understanding of concepts across different researchers and practitioners.

#### Distinguish academic focus on theory from practitioner application

Some participants highlighted the importance of recognizing differences in academic scholars’ priorities versus applied practitioners’ priorities. Participants indicated that while academia can prioritize the development of new theories and models, practitioners should focus on translating these frameworks into practical tools that can benefit individuals and communities. Leveraging the respective strengths of both academia and practice can foster a symbiotic relationship between theory development and practice relevance. Both play crucial, synergistic roles in advancing theory development in positive psychology.

### Criticism 2: problems with measurement and methodology

#### Expand methodologies beyond empiricism and self-report

Participants indicated that positive psychology relies too heavily on empirical data, surveys, and self-report measures to measure and model positive phenomena. It was argued that positive psychology should expand its repertoire of assessment tools to include more projective techniques, free associated narrative interviews, narrative approaches (e.g., storytelling), art, and other creative approaches to assess positive phenomena. These methods can capture the nuances of positive phenomena and provide individuals with alternative means of self-expression. Further, it was stated that researchers should balance the use of objective assessment measures with exploratory approaches, as such is crucial for capturing real-world lived experiences. Emphasizing qualitative methods alongside quantitative measures can offer a more comprehensive understanding of positive phenomena. Participants proposed an array of other solutions like adopting pluralistic approaches to understand multidimensional wellbeing using mixed methods, case studies, storytelling techniques, physiological measures, and participatory community-based research. Further, specific focus should be on exploring means to assess collective positive experiences, in addition to individual mechanisms. Developing methods that capture group dynamics, social connections, and shared well-being can enhance the field’s understanding of positive change at an institutional, community or societal level. Diversifying methodologies can capture positive phenomena more comprehensively while mitigating cultural biases.

#### Promote open science practices and data sharing

Enhancing transparency, documenting failures, and sharing data through open science initiatives can strengthen positive psychology’s credibility. Sharing data, methodologies, and results can contribute to the cumulative growth of knowledge in the field and facilitate critical evaluation. Further, participants indicated that journals should dedicate sections to publish “negative” or non-significant results to counter publication bias and promote transparency. This practice fosters a more balanced representation of research outcomes and allows for a comprehensive evaluation of the field’s progress.

#### Control for social biases and respondent reactivity

Participants stated that positive psychology research suffers from confirmation bias and social desirability biases that undermine objectivity. Using indirect, implicit, and behavioral measures, along with anonymity and participant corroboration, can help control for these limitations. Social desirability should therefore be controlled for in studies to ensure the accuracy and validity of measurements. Implementing strategies to minimize this bias can enhance the reliability of positive psychology research. Finally, participants indicated that researchers should explore respondent reactivity to positive psychology surveys in order to optimize measurement.

### Criticism 3: issues with replication and evidence

#### Enhance ethical science communication and public skepticism

Misrepresentations of positive psychology in media and popular literature have contributed to perceptions of it as pseudoscience. Promoting responsible science communication by the media is crucial to prevent the spread of false claims in the public domain. Further, educating the public about the nature of scientific research and promoting responsible reporting of findings can mitigate misconceptions and enhance public understanding of positive psychology. Building critical thinking skills and fostering skepticism of scientific claims are essential in this process. Developing educational programs for the public that promote critical evaluation of scientific evidence can empower individuals to discern between reputable research and pseudoscience. Finally, participants called for establishing ethical guidelines for science communication and practice within positive psychology to ensure responsible conduct and uphold rigorous standards for science communication. These guidelines can provide a framework for researchers, practitioners, and media professionals to adhere to in their dissemination of information in a responsible manner.

#### Centralizing scientific communication

Participants also emphasized a need to collate and communicate scientific evidence in a comprehensive and accessible manner through a centralized point of communication, such as through professional societies. This centralized approach enables the field to present a unified front and counter misrepresentations or misunderstandings.

#### Actively engage with critics

Participants stated that engaging actively with critics is important to foster a constructive dialog and address concerns. Researchers can develop testable solutions and refine their approaches by understanding the reasoning behind critics’ skepticism. Collaboration with critics can lead to improvements in research methodology, replication, and the overall robustness of positive psychology. Further, it was stated that researchers should also actively test- and communicate the results of claims about pseudoscience within positive psychology. Engaging in rigorous empirical research, addressing critics’ concerns, and disseminating findings transparently can contribute to evidence-based practices and enhance the field’s credibility.

### Criticism 4: lack of novelty and self-isolation

#### Bridge divides through interdisciplinary collaboration

Positive psychology should proactively bridge divides with fields like humanistic psychology and existentialism through interdisciplinary collaboration and mutually informative dialog. Researchers should build on complementary strengths, while identifying unique contributions of their approaches versus potential redundancies.

Clarifying the distinction between positive psychology as an interdisciplinary, horizontal approach and mainstream psychology as a primarily divided vertical approach (e.g., clinical, organizational psychology) was highlighted as being crucial for advancing the field. Participants argued that positive psychology’s unique value proposition lies in its interdisciplinary relevance and applied nature. Positive psychology does not need to be entirely novel; it can provide alternative explanations and offer a more balanced view of psychological phenomena.

Positive psychology provides novel ways of understanding psychological issues through its focus on studying the balance between positive and negative aspects. Exploring the bright side of negative emotions and understanding the dark side of positive phenomena is important for a comprehensive understanding of wellbeing. Recognizing the nuanced interplay between positive and negative aspects contributes to a more holistic approach to human experience.

### Criticism 5: a decontextualized, neoliberal ideology that causes harm

#### Prioritize cultural responsiveness

Applying positive psychology globally requires cultural humility and co-creating contextualized frameworks of positive phenomena and not just exporting Western models to other cultures. Testing measures and theories across cultures is crucial to determine their cross-cultural applicability. Understanding the cultural variations in positive psychological phenomena can help identify universal aspects and context-specific factors. Placing contexts at the forefront of research rather than considering them ex post facto is essential. Learning from qualitative approaches and incorporating cultural considerations from the outset can lead to more ecologically valid findings and prevent decontextualization. Finally, participants argued that emphasizing bottom-up approaches is necessary to contextualize factors within specific cultural and societal contexts. Building theories from the ground up, in collaboration with participants, enables a more nuanced understanding of positive phenomena within different cultures.

#### Adopt social responsibility ethos

Further, participants state that positive psychology should not absolve itself from social responsibilities. Acknowledging the broader societal implications of interventions and addressing potential harm is crucial. Ethical considerations, social justice perspectives, and a focus on the wellbeing of diverse populations should guide positive psychology’s endeavors. Participants also argued that adopting a “do no harm” ethos is important to mitigate unintended negative consequences of positive psychological interventions. Considering the potential risks and negative aspects of interventions, such as the dark side of positive phenomena, can prevent harm and foster a balanced approach to wellbeing.

#### Balance positive and negative aspects

Acknowledging and exploring the negative aspects of positive psychological states, traits, and behaviors is essential for a comprehensive understanding of wellbeing. Examining when an excessive emphasis on positive factors becomes detrimental helps avoid simplistic or one-sided perspectives.

#### Ensure intervention quality

Participants further argued that interventions should be designed on person-intervention fit principles to ensure that interventions are tailored to individuals’ needs, values, abilities, opportunities, and strengths. This personalized approach maximizes the effectiveness of interventions while minimizing potential harm. Further, participants called for formalizing training, establishing accredited training programs, and providing practice guidelines in order to enhance professional standards within positive psychology. These measures promote accountability, ensure competence, and guide practitioners in ethical and evidence-based practice.

### Criticism 6: a capitalistic venture

#### Balance free vs. paid access to promote equity

Participants agreed with critics that the popularization of positive psychology has enabled a profit motive which may limit accessibility to marginalized groups. However, striking a balance between free and paid services can be beneficial to all stakeholders. Companies and practitioners can consider providing freemium versions of apps or tools, allowing individuals to access basic services for free while offering enhanced features or personalized support for a fee. Public-private and academic-industry partnerships can also optimize distribution of positive psychology research for social impact. This could lead to making tools and techniques developed with public funds freely accessible and can democratize access to positive psychology resources. These types of open access initiatives can ensure that research and interventions are available to a broader audience, reducing potential inequalities in access.

## Discussion

**FIGURE 1:**
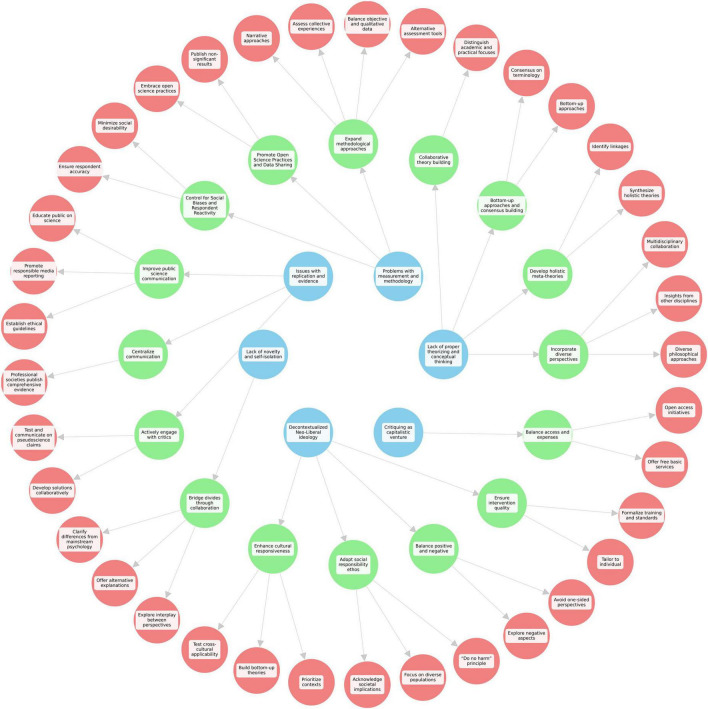
Visualization of the solutions to the criticisms and critiques of positive psychology.

This study aimed to engage positive psychology practitioners and scholars to co-create potential ways to address the main criticisms and critiques of positive psychology through a PAR approach. The results revealed 37 categories of proposed solutions which were organized into 16 broad themes aimed at addressing the six main criticisms of positive psychology (Figure 1). The proposed solutions aligned closely with recommendations by other scholars in the field striving to strengthen positive psychology in its “third wave” by broadening its scope, expanding its methods, by de-contextualizing the development of its theories, and facilitating social-responsible practice (Ivtzan and Lomas, 2016; Lomas et al., 2021; van Zyl and Salanova, 2022). However, some solutions appear idealistic given academic realities. Therefore, thoughtful scrutiny and critical evaluation of these solutions are needed to determine whether such offers viable paths to advance the discipline or if these are no more than idealistic visions.

### Solution 1: lack of proper theorizing and conceptual thinking

Regarding solutions related to the lack of proper theorizing and conceptual thinking, participants primarily argued for incorporating more diverse philosophies, developing integrative or holistic meta-theories, and using inclusive bottom-up approaches into positive psychology. Integrating insights from other philosophical approaches, such as Stoicism and Buddhism, or different theoretical frameworks like complexity theory, can provide alternative explanations for the development of positive psychological phenomena. For example, from Buddhist philosophy an over-emphasis on the strive toward happiness could lead to chronic unhappiness (Levine, 2011) and that happiness comes from focusing on what one can control and accepting things that one cannot control (Ricard, 2014). Further, the Buddhist principles of impermanence, the interconnection between the self and nature, and the reality of suffering can deepen our understanding of human happiness and flourishing (Ricard, 2014). Similarly, Stoicism provides alternative perspectives on virtue ethics, emotional regulation, perspective taking and cognitive distancing which could provide positive psychology with more nuanced perspectives on change, growth, and what constitutes optimal functioning (Macaro, 2018). These suggestion does align with those of other scholars such as Lomas et al. (2021) and Wissing (2022) who also called for incorporating other philosophical traditions into contemporary positive psychology to enhance its theoretical diversity, synthesis, and contextualization. Engaging with diverse philosophical perspectives can offer fresh insights and foster a more inclusive understanding of human flourishing (Van Zyl and Rothmann, 2022; van Zyl and Salanova, 2022).

While commendable, truly incorporating perspectives from different philosophical paradigms is difficult given incompatibilities between their underlying ontological, epistemological and axiological assumptions (Giacobbi et al., 2005). For instance, evolutionary psychology adopts a realist perspective viewing the mind as a set of information-processing modules shaped by natural selection, which directly conflicts with more post-positivist perspectives such as those adopted by positive psychology (Cosmides and Tooby, 2013). Attempting to integrate insights from fundamentally incompatible paradigms risks creating further incoherence within the discipline. A more pragmatic solution may be distinguishing contextual applications of different theories versus forcing integration into a singular framework. Tailored applications of contextualized theories can support the development of a nuanced understanding of positive phenomena while avoiding the need to reconcile irreconcilable metatheoretical assumptions from different paradigm perspectives.

Further, the proposed adoption of bottom-up, inductive approaches to building conceptual foundations for understanding positive phenomena is valuable for promoting inclusion and grounding positive psychology in real-world lived experiences. It emphasizes the development of more holistic approaches to understanding phenomena that’s rooted in local traditions and socio-cultural values (Wissing, 2022). Further, these approaches may allow researchers to capture the nuances and complexities of subjective experiences and can accommodate diverse perspectives, which leads to a more holistic understanding of positive phenomena (Punch, 2013). Similar to the original work of Abraham Maslow, bottom-up approaches could also help with the identification of positive outliers like individuals or communities that demonstrate exceptionally high levels of wellbeing or who are optimally functioning (Punch, 2013). Bottom-up approaches to intervention development also emphasize the importance of collaboration between researchers, participants and communities, which also aids in creating commitment to intervention content and facilitates buy-in (Van Zyl and Rothmann, 2019). This, in turn, increases person-intervention fit and enhances the effectiveness of positive psychological interventions (Schueller, 2014; van Zyl and Rothmann, 2020).

However, only adopting participatory- or bottom-up methods that idealizes organic knowledge creation from “within” communities may also lead to critiques about scientific rigor and bias (Kindon et al., 2007). Balancing scientific rigor with inclusive co-creation is challenging and may lead to new potential issues within the discipline. Punch (2013) stated that only focusing on bottom-up approaches may cause (a) a lack of coherence in theory development, (b) difficulties in generalizing theories to other domains, (c) an incomplete understanding of the phenomena being investigated, (d) bias and increase subjectivity, (e) overfitting (i.e., when theories fit data so well that it fails to generate new information in different contexts), (f) a lack of explanatory power, (g) difficulties in identifying meaningful patterns in relationships and (h) difficulties integrating findings into a cohesive body of knowledge. Similarly, while calls for establishing conceptual consensus or creating “shared definitions” of constructs may have merit for promoting coherence, these universal definitions may not suit the diversity of epistemologies and cultural contexts to which positive psychology aims to generalize (Punch, 2013). A degree of “constructive ambiguity” allowing for pluralistic operationalization of positive constructs may better support global relevance and provide more flexibility to expand theories into new contexts (Klein and Myers, 1999; Tuck and Yang, 2013).

These proposals to diversify and contextualize positive psychology’s theoretical foundations have clear value for enhancing its complexity and inclusiveness. However, integrating radically different perspectives risks creating incoherence, while localized co-creation faces challenges relating to generalizability. A more pragmatic, tailored application of diverse theories may be more viable than seeking an elusive unified theoretical framework for positive phenomena.

Rather than striving for an all-encompassing meta-theory, its perhaps better to proposing ways to deliberately map the connections, overlaps, compatibilities and tensions between diverse theoretical lenses (van Zyl et al., 2023). This would entail closely examining where different philosophical and disciplinary perspectives align and diverge in their assumptions about the nature, determinants and processes underlying wellbeing and optimal functioning. For instance, perspectives on happiness and flourishing that are rooted in hedonic traditions that view happiness as pleasure attainment would have inherent tensions with eudaimonic conceptions focused on self-actualization and meaning-making (Van Zyl and Rothmann, 2014). Similarly, secular psychological frameworks centered on individual agency would conflict with perspectives derived from spiritual philosophies that construe wellbeing as transcending the individual self (Wong, 2011). By systematically mapping these types of points of convergence and divergence, the field could develop a pluralistic model that respects the unique contributions of different perspectives while also acknowledging their boundary constraints (van Zyl et al., 2023). This could help with creating contingencies that can explain when and for whom different theoretical lenses are most applicable. This approach creates a more balanced epistemological platform which integrates multiple, coexisting perspectives that are more geared toward the phenomenological realities they aim to explain (Wissing, 2022). Such a pluralistic theoretical approach may avoid creating forced or even false unifications between theories or philosophical positions. Instead, it could provide a coherence-enhancing framework for productive dialog across different conceptions of positive psychological phenomena. This approach may provide an integrative path forward whilst still preserving the richness of positive psychology’s multidisciplinary roots. Therefore, ongoing pluralistic dialog and conceptual development are imperative as positive psychology evolves as a scientific discipline.

### Solution 2: problematic measurement and methodologies

Proposed solutions for the measurement and methodology criticisms advocate for expanding methodological approaches and measures, the adoption / promotion of open science practices, and controlling for social biases and responded reactivity. Suggestions include incorporating alternative assessment tools (e.g., objective measures/projective techniques), balancing objective assessment methods with more qualitative approaches (e.g., narrative, and arts-based methods), using more mixed-method or qualitative research designs and determining means to assess collective positive experiences (e.g., team engagement). Aligning with the “methodological pluralism” movement, these proposals for greater diversification holds value for generating multifaceted insights and reducing self-report and cultural biases (Giacobbi et al., 2005). These proposals aren’t new, and have been widely discussed by those by both positive psychological scholars (e.g., Lomas et al., 2021; Van Zyl and Rothmann, 2022; Wissing, 2022) and those within the broader domain of general psychology (Nosek et al., 2015). Some progress in this respect have been made and specific suggestions and their value have been discussed elsewhere (c.f. Lomas et al., 2021; Van Zyl and Rothmann, 2022; van Zyl et al., 2023). However, it is important to consider the counterargument for adopting complete methodological pluralism.

When considering the adoption of more mixed method approaches which focuses on combining qualitative and quantitative methods, there is a risk of “incommensurability” given the fundamentally different axiological assumptions both approaches take to knowledge creation (Sale et al., 2002). Mixed method approaches require a careful integration between quantitative and qualitative data sources, where the focus should be on combining such in a meaningful manner. Researchers should avoid prioritizing one type of data over another within mixed-method studies as such could also lead to biases and misinterpretations (Kindon et al., 2007). Further, there is also a risk of interpretation bias associated with incorporating more objective data (e.g., biomarkers) with more subjective data (e.g., self-report measures or qualitative narratives) as researchers may inadvertently favor the former while downplaying the significance of the latter (Pinson and Wolf, 2003). Finally, the subjectivity critiques leveled at qualitative inquiry should also not be ignored (c.f. Punch, 2013).

Further, proposals to publish null results and adopt open science practices also have merit for addressing concerns about publication bias and improving replicability which aligns to calls for reforms within the broader field of psychology (Nosek et al., 2015). Open science practices, such as preregistration, data sharing, and transparent reporting, are instrumental for enhancing transparency, improving scientific rigor and replicability of findings (Efendic and Van Zyl, 2019). Further, by openly publishing null results, positive psychology can foster a more balanced representation of research findings that prevents the perpetuation of publication bias and increase the overall scientific integrity of the field (Nosek et al., 2015). Moreover, adopting these open science practices and principles can help positive psychology shift toward a culture of openness and inclusivity which could create a more collaborative research environment (Efendic and Van Zyl, 2019). It could also encourage researchers to learn from each other’s failures and successes, avoid the duplication of efforts, reduce resource wastage and accelerate the accumulation of evidence which leads to greater theoretical advancements (Nosek et al., 2015).

Despite the value of adopting these practices, there are also a number of pitfalls to consider. The publication of null results may lead to an overrepresentation of inconclusive findings within the literature, potentially diverting researchers’ attention and resources away from more fruitful lines of inquiry. Further, forcing the adoption of open science practices may introduce an extra layer of academic workload as it requires additional time, effort and resources to pre-register studies, preparing data and scripts for open data platforms etc., This could not only strain limited research budgets and time constraints but may increase stress and diver resources away from other critical research initiatives (Veldsman, 2019). Additionally, open science practices may also raise ethical concerns related to data privacy and participant confidentiality which may require the implementation of different safeguards to protect personal information (Miedema, 2022). According to Efendic and Van Zyl (2019), Miedema (2022) and others, open science practices may also (a) disincentivise researchers from engaging in meaningful work, (b) constrain physical and financial resources, (c) reduce motivation for replication studies, (d) overemphasize the importance of statistical significance, (e) lead to the misuse of data or findings, (f) place an extra burden on the already strained peer-review system, and (g) limit the generalizability of non-significant findings. Despite these challenges, we call for a balanced approach toward adopting and implementing open science practices considering the capacity of researchers and the availability of resources.

Suggestions to control for social desirability biases also respond directly to known measurement limitations. However, demand characteristics and confirmation bias are deeply ingrained within psychology that has evades easy solutions. Controlling for social desirability biases in research may involve using indirect or objective assessment measures, piloting new tools or interventions and using well-validated measuring instruments. As stated above, researchers could also employ more double-blind research procedures and use preregistration as a means to manage potential biases (Krumpal, 2013).

### Solution 3: positive psychology is a pseudoscience: poor replication and lacking evidence

Proposed solutions for addressing the pseudoscience criticism emphasize improving public communication about science, actively engaging with critics, and centralizing evidence dissemination. Suggestions include educating the media and the public about responsible science reporting, fostering critical thinking skills, and establishing ethical guidelines for science communication. These solutions directly respond to issues of misrepresentation and exaggerated claims about positive psychology in popular media contributing to perceptions of it as pseudoscience (Fernández-Ríos and Novo, 2012). However, improving the public’s scientific literacy faces systemic challenges, while engaging critics risk drawing more attention to extreme views (Sarewitz, 2004; Toomey, 2023). A more nuanced path may be acknowledging limitations and uncertainties while educating the public about the self-correcting nature of science.

Proposals to actively test and communicate claims of pseudoscience also have merit for addressing unsupported assertions and enhancing credibility through evidence-based rigor. This presents the opportunity to test critics’ ideas and to collaborate with them to not only generate answers to their questions, but also to foster collaboration and buy-in. However, collaboration with critics, while ideal, can be hindered by epistemic tensions (Innes and Booher, 2010). Skepticism often leads to greater entrenchment and polarization rather than creating a situation that is open for discourse (Sá et al., 1999). Finally, centralizing communication via professional associations could assist in consolidating and disseminating evidence in a socially responsible manner. But these risks being perceived as insular “groupthink” as opposed to an independent evaluation and presentation of scientific evidence.

### Solution 4: lack of novelty and self-isolation

Proposed solutions for the novelty criticism advocate for clarifying positive psychology’s distinct value proposition, recognizing its incremental contributions, and exploring positive-negative dialectics. Suggestions to distinguish positive psychology’s interdisciplinary focus from the specializations in mainstream psychology have merit as a means to articulate its unique value proposition (Donaldson et al., 2015). Further, proposals to position positive psychology as complementing, rather than supplanting, mainstream frameworks also make pragmatic sense. Psychology constantly evolves through cycles of thesis, antithesis, and synthesis (Wissing, 2022). Positioning positive psychology as synthesizing humanism’s focus on growth with empirical rigor can be unifying.

However, defining positive psychology’s own boundaries is inherently challenging given the interconnected- or porous nature of the overall discipline (Donaldson et al., 2015) as well as the interdisciplinary nature of its subject matter (van Zyl et al., 2023). For instance, humanistic psychology shares its emphasis on growth, self-actualization, hope, and meaning not only with positive psychology, but with existentialism and cognitive-behaviorism (Waterman, 2013). Further, positive psychological practices like gratitude, mindfulness, and character strengths owe debts to religious and philosophical traditions (Kristjánsson, 2013) and therapeutic approaches fostering positive growth align with both traditional clinical and positive paradigms (Wong, 2011). Clearly, wellbeing and optimal functioning are thus complex phenomena that spans multiple levels and stretch across and between various domains of inquiry. However, several key characteristics can be proposed to help clarify what distinguishes positive psychology from other domains.

First, positive psychology can be characterized by both a preventive and promotive orientation toward mental health and wellbeing which focuses on building strengths and nurturing healthy habits, in contrast to mainstream psychology’s historical emphasis on understanding disorders and managing current challenges (Wissing, 2022). This shifts the field’s central preoccupation to not only understanding and facilitating the generative conditions and processes that enable individuals, organizations, and communities to thrive, but also how these conditions can be used to prevent the onset of psychopathology.

Second, positive psychology can be viewed as an interdisciplinary, or integrative space that synergistically bridges insights from diverse theoretical lenses and disciplines to create more holistic understandings of human flourishing (Van Zyl et al., 2024). While not necessarily generating completely original or new constructs, it could provide pluralistic meta-frameworks that contextualizes complementary perspectives from fields like developmental science, social psychology, organizational behavior, and neuroscience around common wellbeing themes (van Zyl et al., 2023).

Third, positive psychology is an inherently applied discipline that translates its knowledge into practical, evidence-based practices, interventions, and policies. Its iterative cycles of theory-building and interventions distinguish it from purely descriptive or “experiment only” subdisciplines of psychology (Donaldson et al., 2015). This applied, solutions driven focus that presents practical solutions to real-world issues perhaps be one of its key differentiating factors.

Finally, as an evolving discipline, positive psychology’s boundaries doesn’t necessarily have to be rigid and clearly demarcated. It has to be dynamic so that it can be flexible in accommodating new emerging philosophical, cultural and scientific perspectives that could enrich our understanding of human flourishing in different context. Its boundaries could be perpetually re-constituted through collaboration with, between and across different disciplines that are devoted to understanding and nurturing human potential and wellbeing.

### Solution 5: a decontextualized, neoliberal ideology that causes harm

The ideology criticism elicited solutions emphasizing enhancing cultural responsiveness, adopting social responsibility, balancing positive-negative perspectives, and ensuring intervention quality. However, implementing these solutions may be rather complex. Suggestions to test measures cross-culturally intend to ascertain the universality of the constructs and instruments as apposed to determining their cultural specificity. However, positivist, etic instruments developed in Western contexts often pose psychometric and practical problems when implemented in non-Western contexts (Christopher and Hickinbottom, 2008). Collaborating with cultural insiders or locals to co-create emic theories and assessment measures is therefore ideal but often poses challenges to scale across contexts (Nastasi and Hitchcock, 2016). Similarly, when co-creating knowledge with indigenous groups, negotiating insider-outsider dynamics may introduce power imbalances that can subordinate indigenous knowledge to western thinking (Tuck and McKenzie, 2015).

Further, proposals for adopting more qualitative, inductive approaches have merit for countering decontextualization by starting from lived experiences of participants rather than superimposing ideas onto them. As discussed above, adopting more participatory methods face critiques about scientific rigor and bias (Kindon et al., 2007). Standards for ensuring methodological rigor while preserving subjective, cultural voices and lived experiences of the individual remain elusive within the literature. There should thus be a balance between emic sensitivity with etic comparability when adopting such an approach (Nastasi and Hitchcock, 2016).

Recommendations by participants to acknowledge societal implications and to prioritize social justice is commendable and align with positive psychology’s core aims. However, systemic inequities in society and science persist despite well-intentioned efforts to manage such (Prilleltensky, 2008). Transforming entrenched power structures and inequalities remains challenging as its resistant to quick change (Prilleltensky, 2008). Given the Western focus of the majority of positive psychology, individual efforts cannot readily or easily dismantle inherent power structures. A systemic change in required to ensure that marginalized groups are not discriminated against or ignored in the development of new positive psychological theories or approaches.

Finally, suggestions about improving interventions, and practice standards through formalized training, the accreditation of academic programmes and the development of practice guidelines are crucial is prudent. Suggestions to tailor interventions to individuals’ specific needs, values, capabilities and strengths could enhance the person-activity fit, which enhances the effectiveness of the intervention and minimizes potential harm (Schueller, 2014). However, personalizing interventions is not only expensive, but balancing costs while maintaining rigor requires additional skills (Rao and Donaldson, 2015). Not all practitioners possess the skills, competencies, and capabilities required to develop scalable interventions that balance person-activity fit, rigor and the availability of resources. Such necessitates improved training and development. Further, suggestions to formalize training standards and educational requirements to practice as a positive psychological practitioner aim to ensure practitioners are accountable, competent, and well-equipped to disseminate interventions (Rusk and Waters, 2015). However, regulating an emerging, heterogeneous field while allowing for innovation and flexibility is complex. Establishing ethical codes and practice guidelines can enhance socially responsible practice that balances rigor with relevance (Rao and Donaldson, 2015).

### Solution 6: a capitalistic venture

Critics contend that the popularization of positive psychology has enabled profit motives which limit access, exacerbate inequalities and medicalizes positive psychological phenomena. Proposed solutions aim to balance financial sustainability of practice with equitable open access for individuals/clients. However, challenges remain in reconciling these competing aims. Suggestions for hybrid business models that offer basic free access with premium paid features intend to balance the financial viability of platforms and access (van Zyl et al., 2023). However, companies will ultimately prioritize profitability over service, so free versions of applications, tools and interventions are often deliberately limited, restricting lower-income groups to watered-down versions of these resources.

Participants also suggested that publicly funded research should lead to open access distribution of validated tools, techniques and interventions. This democratization of knowledge supports applied progress and access even if profit motives persist within industry (van Zyl et al., 2023). It is, however, important to note that quality open-access publishing and distribution of content has substantial costs and the distribution of such is often prohibitive without private subsidies or permissions (Tennant et al., 2016). Relying on strained public resources alone may not be sustainable or realistic to facilitate the open-access distribution of resources.

### Limitations and recommendations

While this study provides valuable insights into and critical reflection on potential solutions for addressing major criticisms of positive psychology, some limitations should be noted.

The first consideration pertains to the PAR process. While the PAR approach provided an inclusive platform for positive psychology stakeholders to reflect on the field’s criticisms, the process itself had certain inherent limitations worth discussing. The type of sample, being rather homogeneous, and the nature of the topic may have inadvertently shaped the types of solutions proposed (Chevalier and Buckles, 2019). During the discussion, it would seem as though some criticisms resonated more strongly with participants than others and thus garnered more substantiative proposals. For instance, discussions around the theoretical challenges, methodological issues and cultural responsiveness of the field were more richly debated. In contrast, the critiques that related to positive psychology being a profit-driven, capitalistic venture not only generated fewer concrete proposals but also solicited less debate and discussion. This discrepancy likely reflects participants’ own areas of expertise and primary concerns as scholars and practitioners (Chevalier and Buckles, 2019). Further, participants blind spots around broader socio-political and economic forces may have constrained their ability to thoroughly unpack the critique around positive psychology being a profit-driven initiative. The absence of voices from outside the positive psychology community, such as those from critics, entrepreneurs working in the wellbeing space and even the general public could have potentially compounded these blind spots.

Further, the PAR process and the homogenous nature of the sample could have stimulated confirmation bias (Chevalier and Buckles, 2019). This may have biased the results toward optimistic, idealized solutions versus more skeptical assessments of the feasibility of the potential reforms. This confirmation bias may also have led participants to focus on solutions that drives or evolves the field rather than critically dismantling each problematic area. The PAR process coupled with participants’ investment in positive psychology creates an inherent tension when presented with criticisms that challenges conventional positive psychological wisdom and its philosophical foundations. Ultimately, while providing an invaluable contextualized perspective, the PAR process may have created a breeding ground for group think, and thus silenced voices expressing further criticism of the solutions or for proposals which called for radical paradigm shifts (Chevalier and Buckles, 2019). Future research could incorporate a more diverse range of stakeholders, including critics of positive psychology, to generate alternative proposals. This approach may not only present new proposals but could potentially provide a more enriching discussion around the proposals presented in this paper.

Second, the study did not determine the relative importance or viability of the different proposed solutions. Additional priority-setting research could help identify the most critical or actionable solutions which could be used as a roadmap for change. Similarly, gathering perspectives on potential barriers, resources needed, and timelines for implementing solutions could provide a more realistic action plan. Finally, the conventional content analysis provides a descriptive summary of proposals but lacks a deeper critical analysis of the suggested solutions, their merits and potential limitations.

## Conclusion

This critical analysis of the suggestions of the participants highlights the complexities of translating idealized solutions into meaningful progress. While the proposed solutions offer worthwhile food for thought, their wholesale implementation faces barriers related to incommensurable paradigms, institutional constraints, skepticism, epistemic tensions, and global inequities. Sincere engagement with positive psychology’s limitations is crucial. But realistic appraisals of the viability of proposed solutions can complement aspirational brainstorming as a means to advance the field. Nonetheless, this study makes a valuable contribution by tapping insider perspectives on constructive paths forward amidst criticism and controversy. Through collaborative discourse around limitations and potential responses, positive psychology can continue maturing into a more rigorous, culturally responsive, self-reflective, and impactful science in service of human flourishing globally.

## Data Availability

The data analyzed in this study is subject to the following licenses/restrictions: sharing the data publicly was not part of the informed consent process. Requests to access these datasets should be directed to llewellyn101@gmail.com.
